# Association between maternal autistic traits and children’s anxiety among Chinese preschool children in the general population: the chained mediation model of maternal meta-emotion philosophy and children’s emotional instability

**DOI:** 10.3389/fpsyg.2026.1748760

**Published:** 2026-04-17

**Authors:** Jiyou Gu, Yinghua Liu, Yuxin Shan, Hewen Xu, Danning Zhang

**Affiliations:** 1 Faculty of Education, Shandong Normal University, Jinan, Shandong, China; 2 The Second Experimental Kindergarten of Jiaozhou City, Qingdao, Shandong, China; 3 Shandong Mental Health Center, Shandong University, Jinan, Shandong, China

**Keywords:** chained mediating model, children’s anxiety, children’s emotional lability, maternal autistic traits, maternal meta-emotion philosophy

## Abstract

**Background:**

Little research has elucidated the effects of maternal autistic traits (MATs) on children’s anxiety in normal populations, and their underlying mechanisms. The present study aimed to test this relationship and the mediating role of maternal negative meta-emotional philosophy (MEP) and children’s emotional instability among Chinese people.

**Methods:**

This study recruited 590 mother–child dyads. These mothers have no other children with autism, and they completed the Autism Spectrum Quotient, Maternal Meta-Emotion Philosophy, Emotion Regulation Checklists, and the Chinese version of the Spence Children’s Anxiety Scale. The chain mediation model was tested using SPSS software.

**Results:**

(1) There are significantly positive correlations among MATs and children’s anxiety. (2) Children’s anxiety was affected by MATs through 3 different pathways: the mediating role of maternal MEP (dysfunction and noninvolvement emotional philosophy), the mediating role of children’s emotional lability, and the chain mediating role of both MEP and children’s emotional lability.

**Conclusions and limitations:**

This cross-sectional study demonstrates that MATs predict child anxiety through the sequential mediation of mothers’ negative MEP and children’s emotional instability. These findings deepen our understanding of the adverse effects of subclinical autistic traits within the general population. Furthermore, they suggest that early interventions for families with mothers exhibiting high autistic traits should focus not solely on the traits themselves, but on improving maternal MEP. Such a focus would help children develop adaptive emotion regulation strategies, thereby reducing the risk of anxiety. A primary limitation of this study is its cross-sectional design, which precludes causal inferences. Future longitudinal research is needed to clarify the temporal dynamics and long-term effects among these variables.

## 1 Introduction

Autistic traits (ATs) refer to a set of behavioral, cognitive, and personality characteristics that resemble features of autism spectrum disorder (ASD) but occur subclinically in the general population (Bralten et al., 2019). Research indicates that ATs are continuously distributed across the population (American Psychiatric Association, 2013). The existing research has shown that individuals with elevated levels of ATs tend to experience greater emotional dysregulation, display fewer pro-social behaviors, and exhibit lower social competence compared to those with lower ATs (Cetinoglu and Aras, 2022; Dow et al., 2021; Zhao et al., 2019).

Current research on ATs has predominantly focused on their role in predicting cognitive, emotional, and behavioral outcomes among children diagnosed with ASD. In contrast, relatively little is known regarding the influence of parental ATs on children with typical development. With the exception of a few recent studies, this area remains underexplored. For instance, Ronconi et al. (2014) demonstrated that higher parental ATs predict visual attention deficits in infants. Using a longitudinal design, Loncarevic et al. (2021) found that parental ATs were associated with communicative difficulties in children at 6 and 24 months of age. Additionally, Roberta et al. (2021) reported that parental ATs may contribute to impaired statistical learning abilities in infants. It is important to note that significant gaps remain. First, while the effects of parental ATs have been validated in infant populations, their impact on preschool-aged children is still poorly understood. Second, existing studies have primarily examined cognitive developmental outcomes, largely overlooking emotional outcomes and the underlying mechanisms involved.

Anxiety, as the most prevalent mood disorder in early childhood, represents a significant contributor to impaired mental health among children (Polanczyk et al., 2015). Extensive research has demonstrated that anxiety during early childhood predicts a range of adverse outcomes in adolescence, including depression and conduct disorders (Dyer et al., 2019), underscoring its profound implications for both contemporary and long-term development. Consequently, identifying factors associated with childhood anxiety and mitigating its impact hold considerable practical and theoretical importance.

According to ecological system theory (Bronfenbrenner, 1979), the family constitutes the primary microsystem influencing child development. Within this context, maternal personality traits may serve as direct or indirect factors affecting children’s anxiety (Koutra et al., 2017; Pearson et al., 2017). For instance, studies have shown that higher maternal neuroticism (Leonardi et al., 2021) and lower levels of warmth and responsibility (Van Oudheusden et al., 2020) are associated with increased anxiety in children. Notably, ATs have been correlated with specific personality dimensions, including extraversion, agreeableness, conscientiousness, and neuroticism (Robinson et al., 2020). It is therefore plausible to hypothesize that maternal ATs (MATs) may predict anxiety levels in children.

Meta-emotional philosophy (MEP) encompasses parents’ perceptions and attitudes toward their own and their children’s emotions (Gottman et al., 1997; Stettler and Katz, 2014; Shine and Wampler, 1997). Accumulating evidence suggests that negative MEP in mothers may contribute to emotional difficulties in children. Mothers who endorse emotion dysregulation beliefs (EDB) often respond to strong emotions—particularly anger—in less adaptive ways, which can lead to dysfunctional emotional expression in their children (Hooper et al., 2018). These mothers frequently experience overwhelm in the face of negative emotions, both their own and their children’s, and exhibit difficulties in regulating such affect. This may evoke fear and anxiety in children, who also struggle to accurately interpret their mothers’ emotional cues. On the other hand, emotional non-involvement beliefs (ENB) are characterized by a lack of attention to children’s negative emotions, failure to identify the causes of these emotions, or minimal effort to respond supportively (Hu et al., 2017). ENB can impede children’s ability to calm down and manage negative affect, thereby exacerbating emotional dysregulation. Furthermore, it limits opportunities for children to learn effective emotion regulation strategies through parental modeling.

ATs may influence mothers’ MEP. Similar to individuals ASD, individuals with elevated ATs often exhibit deficits in effective emotion regulation strategies (Zhao et al., 2019; Mallise et al., 2020). This may lead them to perceive their own and others’ emotions as unmanageable, resulting in higher scores on EDB. Research indicates that mothers with high EDB experience difficulties in managing their own and their children’s negative emotions, often leading to less constructive emotional expression. This dysregulation can contribute to increased anxiety, depression, and anger in both themselves and their children (Chang et al., 2021). On the other hand, according to the social motivation theory of ASD (Chevallier et al., 2012), social functioning deficits in ASD may stem from reduced social motivation. It is plausible that as MATs increase, mothers may demonstrate less willingness to engage in social and emotional exchanges—particularly in response to their children’s negative emotions—and may be less inclined to communicate with or understand their children’s emotional experiences. This behavioral pattern would likely correspond to higher scores on emotional non-involvement beliefs (ENB). Empirical evidence suggests that maternal ENB can lead children to feel undervalued and misunderstood, potentially triggering emotional issues such as depression and anxiety (Chang et al., 2021). Based on this reasoning, both EDB and ENB are proposed to mediate the relationship between MATs and child anxiety.

MATs may contribute to reduced emotional stability in children. As noted previously, individuals with elevated levels of ATs exhibit emotional characteristics similar to those observed in autism spectrum disorder (ASD), particularly regarding emotion regulation deficits and associated functional impairments. Empirical studies have shown that within the general population, higher levels of ATs are associated with less effective emotion regulation strategies and lower emotional functioning (Zhao et al., 2020). According to the Model of the Socialization of Emotion (Eisenberg et al., 2001), parents’ emotional responses and behaviors serve as templates for children’s own emotion expression and regulation, thereby influencing their emotional and behavioral stability. Similarly, social learning theory posits that children learn to interpret and manage emotions through observational learning, particularly from authority figures such as their mothers. Thus, if a mother demonstrates poor emotional functioning—especially in emotion regulation—her child may adopt similar maladaptive strategies, leading to the development of emotional instability. Research further indicates that deficits in emotion regulation resulting in emotional instability predispose children to emotional disorders such as anxiety (Mennin et al., 2002). Moreover, interventions targeting emotion regulation have been shown to effectively reduce anxiety in children, supporting the notion that stronger regulatory abilities are associated with lower anxiety (Eisenberg et al., 2001). Taken together, these findings suggest that MATs may heighten children’s emotional lability (EL), which in turn increases their vulnerability to anxiety.

A robust association has been observed between maternal MEP and children’s EL. Negative maternal MEP has been found to predict impairments in children’s emotion regulation, resulting in elevated emotional instability, which in turn adversely affects peer relationships, academic performance, and broader developmental outcomes (Ziv and Arbel, 2020). Specifically, mothers who exhibit EDB or ENB not only contribute directly to their children’s negative emotional experiences but are also associated with lower inhibitory control in children, further compromising their emotional regulatory capacities (Castro et al., 2015; Guss et al., 2015). These findings collectively suggest that negative maternal MEP and children’s EL may function as serial mediators in the relationship between MATs and child anxiety.

In summary, this study investigated the relationship between MATs and anxiety in young, typically developing children, as well as the underlying psychologically mechanisms. The study hypothesized that: (1) MATs significantly and positively predict children’s anxiety; (2) negative maternal MEP (EDB, ENB) mediates the relationship between MATs and children’s anxiety; (3) children’s EL mediates the relationship between MATs and children’s anxiety; and (4) negative maternal MEP (EDB, ENB) and children’s EL serve as serial mediators in the relationship between MATs and children’s anxiety.

## 2 Methods

### 2.1 Participants and procedures

Using a cluster sampling approach, the initial sample for this study comprised 603 mother–child dyads with typically developing children aged 4–6 years. After data screening, invalid questionnaires—including those with excessive missing values, random responses, or patterned answers—were excluded. The final analytic sample consisted of 590 dyads, yielding a valid response rate of 97.84%. The mothers ranged in age from 24 to 52 years (M = 32.15, SD = 7.11). In terms of socioeconomic status, 22.15% of mothers had completed high school, while 63.18% had attained a college degree or higher. None of the participating mothers had a child diagnosed with autism spectrum disorder (ASD). The children (286 boys, 304 girls) had a mean age of 5.78 years (SD = 0.89), and approximately 26.77% were only children.

Participants were recruited from four kindergartens located in Jinan and Qingdao, two major cities in Shandong Province, Eastern China. Mothers provided informed consent through the kindergartens and were invited to attend educational lectures on family education and child development. Following the lectures, participants completed the research questionnaires. Each mother received a gift valued at approximately ¥30 as compensation for their time. The study protocol was reviewed and approved by the Institutional Review Board of Shandong Normal University.

### 2.2 Measures

#### 2.2.1 MATs

MATs were measured using the mandarin version of the Autism Spectrum Quotient (AQ) questionnaire, which is a self-administered questionnaire for measuring ATs in adults with normal intelligence (Zhang, 2016). The scale consists of five dimensions, namely social skills, attentional shifts, attention to detail, verbal communication, and imagination, scored on a 4-point scale and reported by mothers. The total score for each dimension is used as the AQ score, with higher scores indicating higher levels of ATs. The scale has high reliability and validity in existing studies (Meng et al., 2021). The Cronbach’s 𝛼 coefficient of the total scale in this study was 0.86.

#### 2.2.2 Maternal MEP

The MEP scale was used to measure mothers’ EDB and ENB. All components are scored on a 6-point scale. The scale was created using the paradigm for parental MEP proposed by Ye et al. (2005) and was verified in a population of Chinese parents (Liang et al., 2016). Mothers completed the 20-item measure of ENB (20-item) and EDB (8-item). In the present study, internal consistency for the ENB (Cronbach’s 𝛼=0.88) and for EDB (Cronbach’s 𝛼=0.92) was excellent.

#### 2.2.3 Children’s EL

The Emotion Regulation Checklists (ERC) (Shields and Cicchetti, 1997) is a parent-report measure designed to assess a child’s ability to manage and regulate emotions. The Emotion Regulation and Lability subscales comprise the two sets of 24 items that make up the ERC. The emotional lability dimension assesses children’s tendency to experience rapid changes in mood and the frequency of negative emotional expression and was used in this study. This scale has good internal consistency in established studies (Zhu et al., 2020). Cronbach’s alpha of EL in the present study was 0.91.

#### 2.2.4 Preschool children’s anxiety

The Chinese version of the Spence Children’s Anxiety Scale for parents (SCAS-P) (Spence, 1998; Wang et al., 2016) was used to measure mothers’ reports on children’s anxiety. The scale has 44 items, 6 of which are positively worded filter items and not be scored. Children’s anxiety was obtained on a four-point scale (from 0 “never” to 3 “always”) by their mothers. This scale uses the average of all item scores as the preschooler’s anxiety score, with higher scores indicating higher levels of anxiety. The scale has high reliability and validity in existing studies (Ding et al., 2022). The Cronbach’s 𝛼 coefficient for the total scale in this study was 0.90.

### 2.3 Data analysis

The mediation analyses were conducted using Model 6 of the PROCESS macro for SPSS (version 3.3) (Hayes, 2015), which is suitable for testing models with multiple mediators. Indirect effects were examined using a bias-corrected bootstrapping approach with 5,000 resamples. A significant mediation effect was determined if the 95% bias-corrected confidence interval did not include zero. Children’s gender and age were included as covariates in all models. Although parental education level and family income were initially considered as potential covariates, they were excluded from the final analyses due to excessive missing data (more than 20% of cases). A *post-hoc* analysis on the subsample with complete SES data confirmed that the core mediation findings remained significant after controlling for parental education and family income, indicating no confounding effect of socioeconomic stressors.

## 3 Results

### 3.1 Testing for common method bias

Twenty-nine factors with eigenvalues larger than one were identified by Harman’s single-factor test. Relative to the suggested cutoff point of 50%, the first component only accounted for 12.16% of the total variances. Therefore, we may conclude that this study’s common method bias is not very problematic.

### 3.2 Preliminary analysis

A multivariate analysis of variance (MANOVA) of 2 (child gender: boy and girl) × 3(child age: 4-, 5-, and 6-years old) was conducted for children’s anxiety. The results revealed that the main effect of child gender, the main effect of child age, and the interaction effect of child gender and age (*ps*> 0.05) for children’s anxiety were not significant.

Table 1 shows the means, standard deviations (SD), and the Pearson correlations of each study variable. There was a positive correlation between all hypothetical pairwise variables (r = 0.21–0.80, *p*<0.01).

**Table 1:** Means, standard deviations, and correlations of the study variables (*N* = 590).

Variable	*M*	*SD*	1	2	3	4	5
1. MATs	2.32	0.20	1				
2. Mothers’ EDB	3.49	0.59	0.21^**^	1			
3. Mothers’ ENB	3.42	0.65	0.22^**^	0.80^**^	1		
4. Children’s EL	2.93	1.08	0.26^**^	0.28^**^	0.27^**^	1	
5. Children’s anxiety	2.05	0.50	0.32^**^	0.23^**^	0.25^**^	0.44^**^	1

M, mean; SD, standard deviation. ^**^p < 0.01.

### 3.3 Test of mediation

**Figure 1:**
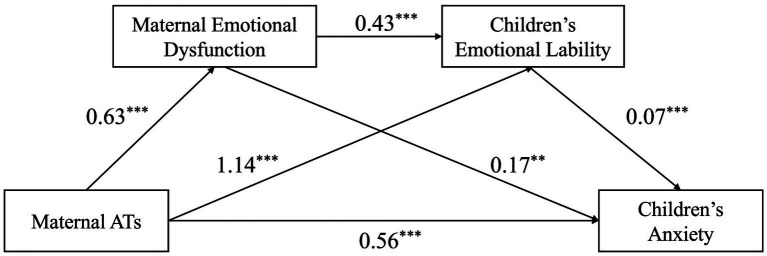
The pathway of the mediation model A. ^**^p < 0.01; ***p < 0.001.

**Figure 2:**
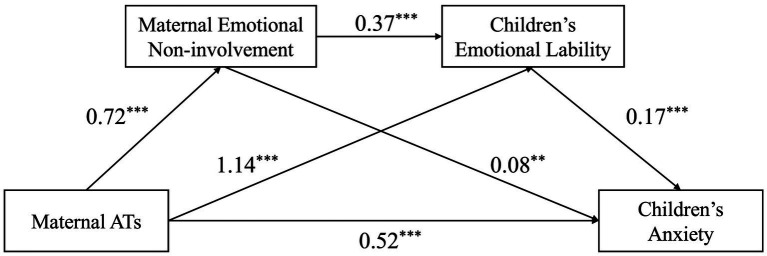
The pathway of the mediation model B. ^**^p < 0.01; ***p < 0.001.

The relationship between MATs and children’s anxiety was examined, with maternal EDB/ENB and children’s EL included as mediators. Figures 1, 2, along with Table 2, present the chain mediation model and its path coefficients. The results indicated that MATs were significantly associated with children’s anxiety, maternal EDB/ENB, and children’s EL. Maternal EDB/ENB was positively associated with children’s EL, which in turn was positively associated with children’s anxiety. When both mediators were included in the model, MATs showed a positive indirect effect on children’s anxiety through these pathways. The mediating effects of maternal EDB/ENB and children’s EL accounted for 17.86%/13.79% in each of the two models. The 95% confidence intervals for all paths excluded zero, supporting the presence of chain mediation. The results are summarized in Figures 1, 2 and Table 3.

**Table 2:** Regression analysis of the relationship between MATs and children’s anxiety.

Outcome variable	Predictive variable	*R*	*R^2^*	*F*	*β*	t
Model A						
Maternal EDB	MATs	0.21	0.05	27.55	0.63	5.25^***^
Children’s EL	MATs	0.34	0.12	39.25	1.14	5.28^***^
	Maternal EDB				0.43	5.84^***^
Children’s anxiety	MATs	0.50	0.25	64.37	0.56	5.59^***^
	Maternal EDB				0.07	2.18^**^
	Children’s EL				0.17	9.58^***^
Model B						
Maternal ENB	MATs	0.22	0.05	29.18	0.72	5.40^***^
Children’s EL	MATs	0.34	0.12	38.14	1.14	5.27^***^
	Maternal ENB				0.37	5.67^***^
Children’s anxiety	MATs	0.50	0.25	65.53	0.52	5.50^***^
	Maternal ENB				0.08	2.71^**^
	Children’s EL				0.17	9.51^***^

**Table 3:** Comparisons of the bootstrap results of the serial mediation Model A and B.

Path	Estimates	SE	95%CI lower	95%CI upper	Relative mediation effect
Model A					
Total effect	0.81	0.10	0.61	0.99	100.00%
Direct effect	0.53	0.09	0.34	0.71	65.43%
Indirect effect	0.28	0.05	0.20	0.37	34.57%
MATs → Maternal EDB → Children’s anxiety	0.04	0.03	0.002	0.09	14.29%
MAT→ Children’s EL → Children’s anxiety	0.19	0.04	0.12	0.27	67.86%
MATs → Maternal EDB → Children’s EL → Children’s anxiety	0.05	0.01	0.02	0.08	17.86%
Model B					
Total effect	0.81	0.10	0.61	0.99	100.00%
Direct effect	0.52	0.09	0.33	0.70	64.20%
Indirect effect	0.29	0.05	0.21	0.38	35.80%
MATs → Maternal ENB → Children’s anxiety	0.06	0.02	0.01	0.11	20.69%
MATs→ Children’s EL → Children’s anxiety	0.19	0.04	0.12	0.27	65.52%
MATs → Maternal ENB → Children’s EL → Children’s anxiety	0.04	0.01	0.02	0.07	13.79%

## 4 Discussion

The present study established a chain mediation model to examine the predictive effect of MATs on child anxiety and its underlying mechanisms. The results demonstrated that MATs significantly and positively predicted child anxiety, with maternal MEP (EDB/ENB) and children’s EL serving as serial mediators in this relationship.

### 4.1 The relationship between maternal ATs and children’s anxiety

The direct association between MATs and children’s anxiety has been demonstrated in the present research. While previous research has not explicitly examined this relationship, some studies have suggested that elevated ATs in the general population may adversely affect the mental health of family members (Brett and Maybery, 2022; Cruz et al., 2013; Ingersoll and Wainer, 2014). The current findings offer empirical support for this proposition.

The positive prediction of children’s anxiety by MATs may be attributed to the cognitive profile shared between AT and ASD (Warrier et al., 2019; Bralten et al., 2018; De Groot and Van Strien, 2017). Elevated levels of AT have been associated with reduced cognitive flexibility (Saure et al., 2022), impairing the ability to shift attentional goals and adaptively respond to novel situations. Consequently, individuals with high AT may interpret ambiguous environmental information in a negative manner, thereby increasing anxiety in both themselves and their family members—including their children (Gökçen et al., 2014). Specifically, compared to mothers with lower AT, those with high AT are more likely to perceive their surroundings as threatening or unsafe. This interpretation is often transmitted to their children, who may similarly learn to view ambiguous situations negatively and perceive their environment as insecure. This shared cognitive-emotional pattern ultimately contributes to elevated anxiety levels in the children.

### 4.2 The independent mediating effects of maternal MEP and children’s EL

The current findings revealed that maternal MEP (EDB/ENB) mediated the association between MATs and childhood anxiety. More specifically, high levels of MATs were associated with increased endorsement of EDB and ENB among mothers, which in turn predicted elevated anxiety symptoms in their children.

This phenomenon may be attributed to deficits in emotional functioning and socialization associated with ATs. Studies conducted in general populations have shown that individuals with high levels of AT exhibit functional emotional deficits similar to those observed in ASD—particularly in the experience and regulation of emotions—even when overall social functioning remains unaffected (Cai et al., 2019; Zhao et al., 2020; Shannon et al., 2020). On the one hand, MATs positively predicted EDB. This may be explained by the tendency of mothers with higher AT levels to express more negative emotions (e.g., anxiety and depression; Liew et al., 2015), coupled with a reduced capacity to manage their own emotional experiences and employ adaptive regulation strategies. Such individuals often experience overwhelm in the face of negative emotions, resulting in elevated EDB scores (Cai et al., 2019; Boily et al., 2017; Zhao et al., 2020; Shannon et al., 2020). On the other hand, MATs also positively predicted ENB. According to the social motivation model of ASD, deficits in social motivation underlie impairments in social attention, theory of mind, and interpersonal functioning in ASD (Chevallier et al., 2012). Empirical studies further indicate that individuals with ASD show reduced preference for social stimuli compared to neurotypical populations (Sasson et al., 2012). Given the phenotypic similarities between high AT and ASD, it is plausible that mothers with elevated AT exhibit diminished social motivation, leading to reduced engagement in interpreting and regulating their children’s emotions during interactions. This decreased motivational involvement may consequently contribute to higher ENB scores.

The predictive effect of maternal EDB and ENB on children’s anxiety is consistent with existing research. When mothers exhibit poor control over their own emotional expression—particularly through emotionally unstable and variable behaviors—children may experience distress, leading to increased behavioral issues and higher levels of negative emotions such as anxiety and depression (Merchant et al., 2019). Furthermore, if mothers fail to actively guide their children in appropriately expressing and regulating emotions, but instead adopt a laissez-faire approach, children may perceive this as maternal inattention or neglect. Such perceptions can contribute to the development of anxiety and other emotional difficulties (Liang et al., 2016).

The present study identified a mediating effect of children’s EL in the relationship between MATs and child anxiety, indicating that higher levels of MATs were associated with increased EL in children, which in turn contributed to greater anxiety.

As previously noted, elevated ATs are linked to impairments in emotional functioning similar to those seen in ASD, particularly in emotion regulation and management (Cai et al., 2019). Consequently, higher MATs may reduce mothers’ ability to regulate their own emotions, leading to diminished emotional stability. Aligned with the Emotional Socialization Model (Eisenberg et al., 2001) and Social Learning Theory (Bandura and Jeffrey, 1973), children acquire emotion-regulation strategies primarily through observation and imitation of their mothers. Given mothers’ central role in child-rearing, their emotional behaviors act as a primary model for children’s emotional development. When mothers display difficulties in regulating emotions, children may inadequately learn effective emotional coping strategies, thereby fostering their own emotional instability.

Emotional stability constitutes a core aspect of effective emotion regulation. Children with underdeveloped regulatory skills who also lack appropriate models of emotional socialization—especially from their mothers—are more likely to experience chronic emotional instability. Such persistent dysregulation may lead to elevated anxiety, frequently arising from the child’s perceived inability to control their emotional experiences.

### 4.3 The chain-mediated effect of maternal MEP and children’s EL

This study revealed that mothers’ negative MEP, including EDB and ENB, together with children’s EL, functioned as serial mediators between MATs and children’s anxiety. These results align with emotional socialization theory, emphasizing that maternal beliefs and attitudes about emotions play a significant role in shaping children’s psychological development (Eisenberg et al., 2001). Distinct dimensions of MEP differentially influence children’s emotional functioning. For instance, mothers who adhere to emotion dysregulation beliefs tend to have children with lower emotional stability (Merchant et al., 2019). These children often acquire maladaptive emotional behaviors, such as poor emotional control, from mothers with high levels of ATs, thereby increasing their own emotional instability and susceptibility to anxiety.

Similarly, maternal ENB was associated with greater emotional instability in children. This relationship may originate from insufficient guidance in emotion regulation, including inadequate instruction in managing emotions or absence of appropriate boundaries for emotional expression, ultimately leading to dysregulated affect and reduced emotional well-being.

### 4.4 Limitations and future research directions

The current study has several limitations. First, although a cross-sectional design was employed to explore the relationship between MATs and children’s anxiety and its potential mechanisms, this approach does not allow for causal inferences. Future research should adopt longitudinal or experimental designs to examine causal pathways and potential dynamic changes in this relationship over time. Additionally, the use of maternal reports for all measures may introduce informant bias, as mothers with higher MATs may perceive or report child emotions differently. Future studies should incorporate multi-informant data (e.g., fathers, teachers) to enhance validity. Second, the study relied primarily on maternal reports of preschool-aged children. Expanding the age range of children and incorporating self-report measures in future studies would provide more comprehensive and developmentally nuanced data. Third, previous research indicates that ATs are more prevalent in males than females (Ronald et al., 2008; Pisula et al., 2013). As paternal involvement in child-rearing continues to increase, future studies should also investigate the influence of fathers’ ATs on children’s mental health and the underlying mechanisms, thereby offering a more complete understanding of parental contributions in this context. Finally, maternal general psychopathology (e.g., anxiety, depression) was not assessed. Given the overlap between autistic traits and neuroticism or emotional distress, the observed effects may partly reflect broader maternal mental health factors. Future research should include measures of maternal psychopathology to examine the specificity of autistic traits.

## Data Availability

The original contributions presented in the study are included in the article/Supplementary material, further inquiries can be directed to the corresponding author.
